# 

**Published:** 1880-08

**Authors:** 


### Table of Contents.

### ORIGINAL COMMUNICATIONS.

The Metric System. By T. W. O'Kelly, M.D., Conyers, Ga......... 257

### REPORTS OF SOCIETIES.

Transactions of the Obstetrical Society of Cincinnati.................. 259
Detroit Academy of Medicine.................................................. 267

### SELECTIONS.

On the Management of the Uterus after Porturition........................ 270
Prof. Clay's New Cancer Cure.................................................. 284
Suppurtive Inflammation of the Middle Ear.................................. 288
Endocarditis: Its Causes and Differential Diagnosis from the Pericarditis.................................................................. 297
Addison's Disease........................................................................ 304

### EDITORIAL.

Tannerism...................................................................... 310
Short-Comings of the Journal................................................ 311
Meteorological Report for July............................................. 312

### EXCERPTA.

A New Remedy for Hydrophobia.............................................. 313
Are Dilatation Instruments Necessary in Gynecological Practice?... 313
Vaginal Hysterotomy.............................................................. 314
A Cure for Snake Bite............................................................ 314
Vulvitis Infantalis.................................................................. 315
Iodoform in Nasal Catarrh and the Treatment of Wounds............... 315
Boracic Acid Injection of Gonorrhœa....................................... 316
Congenital Amaurosis Spontaneously Cured on the First Appearance of the Mestrual Function.................................................... 316
Glycerine Cement.................................................................. 317
Vitality of the Diphtheria Poison............................................. 317
Another Antidote to Arsenic.................................................... 318
A Perfect Solution of Salicylic Acid......................................... 318
Albumen in the Urine.............................................................. 318
The Metric System.................................................................. 320
Disguising the Taste of Epsom Salts......................................... 320

